# Reactive nodular fibrous pseudotumor involving the gastrointestinal tract and mesentery: A case report and review of the literature

**DOI:** 10.3892/ol.2015.2882

**Published:** 2015-01-16

**Authors:** FEI YAN, YANLI MA, JIANHAI SUN, PENGCHENG ZHU

**Affiliations:** 1 Department of Oncology, Zhongshan Hospital of Hubei Province, Wuhan, Hubei 430000, P.R. China; 2 Institute of Pathology, Tongji Hospital, Tongji Medical College, Huazhong University of Science and Technology, Wuhan, Hubei 430030, P.R. China

**Keywords:** reactive nodular fibrous pseudotumor, gastrointestinal tract, mesentery, differential diagnosis

## Abstract

Reactive nodular fibrous pseudotumor (RNFP) is a tumor-like lesion that is characterized by reactive fibroblast/myofibroblast proliferation within collagenic hyalinized stroma, due to its association with injury or inflammation. The current study describes the case of a 60-year-old female with a history of abdominal surgery and abdominal pain. Upon laparoscopy, multiple nodules attached to the outer layer of the colon and mesentery were identified, and therefore, complete surgical excision was performed. Macroscopically, the nodules were well-circumscribed, firm, tan-white in color and ranged in size between 2.0–10.0 cm at the greatest dimension. Microscopically, the nodules were composed of spindle and stellate cells in a dense collagenic hyalinized background with sparse lymphocytic infiltration. Immunohistochemical analysis demonstrated positive staining for vimentin, smooth muscle actin and cluster of differentiation (CD) 117, and focally-positive keratin staining with AE1/AE3; however, no staining was observed for gastrointestinal stromal tumor 1, CD34, S-100, anaplastic lymphoma kinase or β-catenin. Therefore, it was proposed that the lesion may be most accurately described as an RNFP. The current study reports a rare case of RNFP, emphasizing its histopathological features and differential diagnoses to promote an improved and broader understanding of this poorly understood condition.

## Introduction

Reactive nodular fibrous pseudotumor (RNFP) is described as a rare benign tumor-like lesion (1–3), which was first reported by Yantiss *et al* in 2003 (1). According to the limited literature, RNFP predominantly occurs in the gastrointestinal (GI) tract and peritoneal regions with a male predominance (male/female ratio, 14/5) in adults. Macroscopically, it appears as a single mass or multiple nodules attached to the outer layer of the bowel wall (3), mesentery or omentum (2). Microscopically, the lesion is composed of spindle or stellate cells resembling fibroblasts/myofibroblasts enmeshed in a collagenous matrix, which is typically hyalinized or keloidal in nature (1,4). The fact that the lesion often presents with multiple intra-abdominal masses evidently causes clinical concern for malignancy, and complete resection remains a routine treatment. RNFP shows a good prognosis without signs of recurrence or metastasis, no cases of RNFP recurrence or metastasis have been reported in the literature. The present study reports and analyzes a case of RNFP involving the mesentery and greater omentum in a 60-year-old female patient, with the aim of improving the characterization of RNFP, and identifying distinguishing features of the lesion to improve its differential diagnosis from other neoplasms and non-neoplastic lesions involving this anatomical region. The present study was approved by the ethics committee of Zhongshan Hospital of Hubei Province (Wuhan, China) and written informed consent was obtained from the patient.

## Case report

In September 2012, a 60-year-old female patient presented to the Department of Oncology, Zhongshan Hospital of Hubei Province (Wuhan, China) with abdominal pain that had gradually developed over six months following abdominal surgery for leiomyoma of the uterus. Upon laparoscopy, multiple nodules were identified throughout the mesentery, greater omentum and serosal surface of the colon, ranging in size between 2.0–10.0 cm at the largest dimension. Due to the presence of multiple nodules diffused throughout the abdominal cavity, the lesion was diagnosed as a metastatic malignant tumor. Thus, a partial colectomy was performed and all masses were resected prior to macroscopic, histological and immunohistochemical examination of the specimens.

The resected specimens were fixed in 10% formalin, embedded in paraffin, sectioned and stained with hematoxylin and eosin in accordance with routine procedures, and immunostaining was performed on 4-μm-thick sections using the standard avidin-biotin complex technique. A panel of antibodies (Table I) was used to evaluate the tumor samples for the presence of smooth muscle [Desmin and smooth muscle actin (SMA)], fibroblastic/myofibroblastic (SMA), schwannian (S-100) and epithelial cell (AE1/AE3) differentiation, and various immunohistochemical markers typically expressed in GI stromal tumors [GIST; discovered on GIST (DOG) 1, cluster of differentiation (CD) 117, CD34], inflammatory myofibroblastic tumors [anaplastic lymphoma kinase (ALK)], aggressive fibromatosis (β-catenin) and immunoglobulin (Ig) G4-associated disease (IgG4, IgG).

Sections of healthy colon tissue (Maixin Bio, Fuzhou, China) served as the positive controls for the expression of vimentin, SMA, desmin, AE1/AE3, S-100, DOG1, CD34 and CD117, and an ALK-positive anaplastic large cell lymphoma cell line (Maixin Bio) served as the positive control for ALK staining. Negative controls were performed by replacing the primary antibodies with saline.

**Figure 1:**
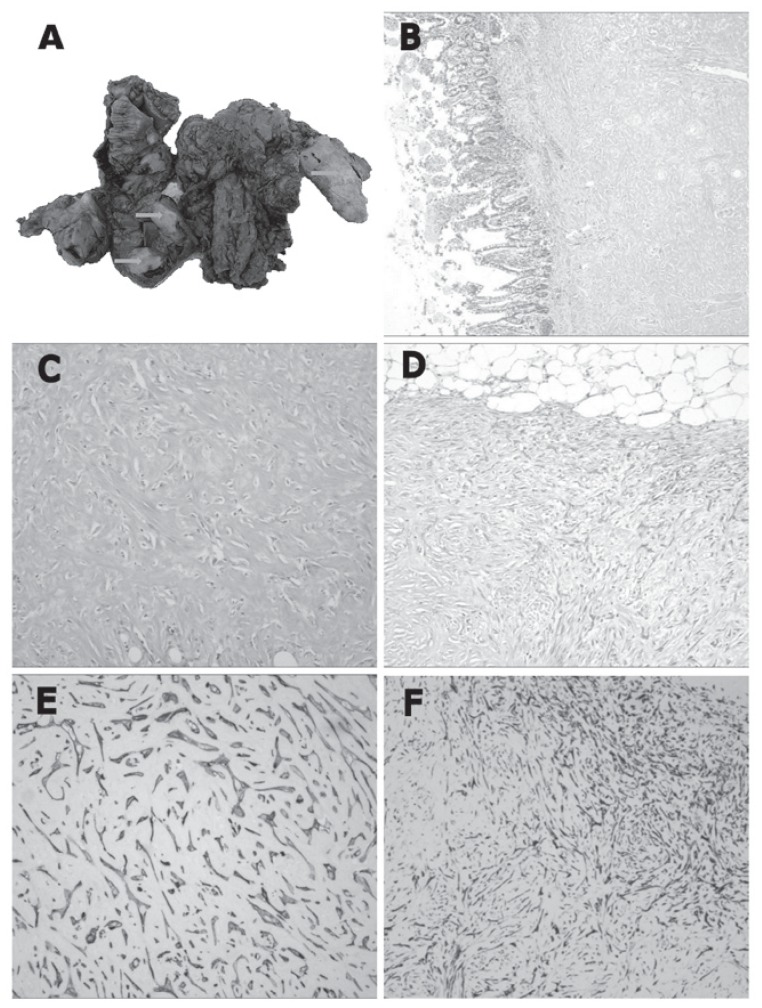
(A) Macroscopic image of the surgical specimen demonstrating multiple tan-white nodules (arrows) of varying sizes attached to outer layer wall of the colon and mesentery. (B) Reactive nodular fibrous pseudotumor (RNFP) of the colon infiltrating and replacing the muscularis propria and encroaching on the submucosa (magnification, ×100). (C) RNFP were composed of proliferative spindle and stellate cells resembling fibroblasts/myofibroblasts in a dense collagenic hyalinized background (magnification, ×400), with (D) specific RNFP nodules containing a more cellular peripheral zone composed of active fibroblasts. (magnification, ×200). (E) Diffuse strong immunohistochemical staining for smooth muscle actin (magnification, ×400) and (F) focal AE1/AE3 expression, particularly in the peripheral cellular zone (magnification, ×200; hemaoxylin and eosin staining).

Macroscopically, the specimens were well-circumscribed, rubbery to hard, white-tan-colored nodules ranging between 2.0–10.0 cm at the largest dimension, while the cut surface of the lesions was solid and homogeneous (Fig. 1A). The nodules predominantly involved the outer layer of the colon, mesentery and omentum, but were partially associated with the transmural extension of the colon. Microscopically, the nodules were composed of sparse wavy spindle or stellate cells within hyalinized keloid-like collagen (Fig. 1B and C), although certain nodules contained a more cellular peripheral zone composed of active fibroblasts (Fig. 1D) or were surrounded by an inflammatory infiltrate of mononuclear lymphoid cells.

Immunohistochemically, the spindle or stellate cells were positive for vimentin, SMA (Fig. 1E) and CD117, focally positive for AE1/AE3 (Fig. 1F) and desmin expression, and demonstrated no DOG1, CD34, S-100 or β-catenin expression. In addition, high counts of IgG4-positive plasma cells were not detected and the ratio of IgG4/IgG-positive cells was determined as <40%.

The patient was regularly followed up, and at 12 months post-resection the patient remains alive and in good health with no evidence of recurrence or metastatic disease. The follow-up continues at approximately eight-week intervals at present.

## Discussion

RNFP was initially described in a series by Yantiss *et al* (1), and is characterized by fibroblastic/myofibroblastic spindle or stellate cells with a hyalinized collagenous background and a lack of atypia and mitosis. RNFP is considered to be a reactive benign lesion that is associated with previous surgical procedures or inflammatory disorders (4). Immunohistochemically, the spindle cells are typically positive for vimentin, SMA, desmin and AE1/AE3 expression, and demonstrated no DOG1, CD34 or S-100 expression. Previous observations of CD117 and AE1/AE3 expression in RNFP (1,4) appear to conflict with the results of the present study, which identified focal positive CD117 and peripheral cellular zonal AE1/AE3 staining. Yantiss *et al* (1) observed CD117 expression in four out of five cases of RNFP, while Daum *et al* (4) confirmed no CD117 expression among eight cases of RNFP. However, analysis of the relevant literature indicates that these conflicting observations may be the result of methodological differences (Table II), such as different antibody clones or antigen retrieval details. Furthermore, the immunohistochemical profile of the present patient was similar to the characteristic fibroblastic/myofibroblastic differentiation of RNFP (5). Considering the specific marker expression profiles of RNFP spindle cells, including the coexpression of vimentin and low molecular-weight cytokeratin, determined by AE1/AE3 expression, it has been proposed that the cells may be derived from multipotential subserosal cells (4).

Cases of RNFP arising within the abdominal cavity (6,7), including the GI tract (3), mesentery and retroperitoneum, are rare but important due to their propensity to be misdiagnosed as more aggressive types of tumor, such as metastatic malignant neoplasm, primary GIST or inflammatory myofibroblastic (IMF) tumor. The case presented in the present study was initially suspected to be a metastatic malignant tumor due to the presence of multiple diffuse nodules within the abdominal cavity. However, histopathological analysis of the lesion determined rare mitotic figures and no nuclear atypia or necrosis. Based upon these clinical and histopathological features, it is proposed that the lesion was a cohesive case of distinct tumor-like fibroblastic/myofibroblastic proliferation that represents an acute post-operative response to surgery.

A recent case series described four patients with RNFP of the GI tract that possessed abundant IgG4-positive plasma cells, indicating that RNFP may form part of the IgG4-associated disease spectrum in specific cases (8,9). However, in the present case, high counts of IgG4-positive plasma cells were not observed and the ratio of IgG4/IgG was <40%.

Certain spindle cell lesions arising from the GI tract should be distinguished from RNFP upon diagnosis. GIST, a common mesenchymal tumor in this anatomical region (10), is typically more cellular than RNFP and is immunopositive for DOG1, CD117 and CD34 (11,12). Although immunopositivity of CD117 and CD34 are the most valuable factors in the diagnosis of GIST, previous studies have demonstrated that DOG1, compared with CD117, may be a more specific and sensitive marker for GIST (13), particularly when RNFP and GIST are each positive for CD117 expression. In addition, calcifying fibrous pseudotumor (14,15), which is generally more cellular than RNFP, including a mixture of granulocytes, plasma cells and infiltrating lymphocytes, frequently presents with psammomatous or dystrophic calcifications and is typically positive for CD34, but exhibits no SMA or desmin expression. Furthermore, IMF (16,17) is another rare tumor that may be located in the mesentery or the retroperitoneum. IMF is a hypercellular tumor composed of loosely arranged fascicles of plump spindle cells with abundant, densely eosinophilic cytoplasm enmeshed within a variably collagenous, edematous or myxoid stroma. Additionally, IMF contains an active inflammatory infiltrate composed of plasma cells and lymphocytes that are closely associated with the tumor cells, in contrast to the mononuclear cell infiltrate of RNFP, which is typically sparse and patchy. Immunohistochemically, ALK-positive staining may also facilitate in the differential diagnosis of RNFP (18). Therefore, metastatic tumors in GI tract may be distinguished from RNFP by patient history and histopathological features.

Non-neoplastic spindle cell lesions that should also be distinguished from RNFP include aggressive fibromatosis (19), nodular fasciitis (20) and sclerosing mesenteritis (21). Thus, RNFP may be distinguished from mesenchymal lesions of the abdomen and GI tract by clinicopathological features and biological potential.

To conclude, RNFP is a post-operative or post-inflammatory lesion that is increasingly recognized in the differential diagnosis of primary and metastatic GI tumors. Therefore, it is important to differentiate RNFP from similar lesions with more aggressive phenotypes, as RNFP may be managed definitively with local resection and surgical follow-up.

## Figures and Tables

**Table I:** Antibodies and dilutions used in the evaluation of reactive nodular fibrous pseudotumor.

Antibody	Dilution	Supplier
Vimentin	1:20	Dako
AE1/AE3	1:20	Dako
CD117	1:40	Dako
SMA	1:100	Dako
DOG1	1:50	Leica Biosystems
CD34	1:20	Dako
Desmin	1:140	Dako
S-100	1:1000	Dako
ALK	1:10	Dako
β-catenin	1:400	Dako
IgG4	1:500	Leica Biosystems
IgG	1:250	Zymed

For all antibodies, heat-induced antigen retrieval was used to improve staining prior to immunohistochemistry. CD, cluster of differentiation; SMA, smooth muscle actin; DOG1, discovered on GIST 1; ALK, anaplastic lymphoma kinase; IgG, immunoglobulin G.

**Table II:** Summary of the reported cases of reactive nodular fibrous pseudotumor.

First author (reference)	Year	Cases, n	Positive expression, protein (n)
Yantiss *et al* (1)	2003	5	Vim (5); CD117 (4); SMA (3); CD34 (0); CK (0); ALK (ND)
Daum *et al* (4)	2004	8	Vim (7); CD117 (0); SMA (8); CD34 (0); CK (6); ALK (ND)
Saglam *et al* (2)	2005	1	Vim (1); CD117 (ND); SMA (1); CD34 (1); CK (1); ALK (ND)
Gauchotte *et al* (7)	2009	1	Vim (1); CD117 (0); SMA (1); CD34 (0); CK (1); ALK (0)
McAteer *et al* (6)	2012	1	Vim (1); CD117 (0); SMA (1); CD34 (0); CK (1); ALK (0)

Vim, vimentin; CD, cluster of differentiation; SMA, smooth muscle actin; CK, cytokeratin; ALK, anaplastic lymphoma kinase; ND, not done.
